# Fractional Modeling of the AC Large-Signal Frequency Response in Magnetoresistive Current Sensors

**DOI:** 10.3390/s131217516

**Published:** 2013-12-17

**Authors:** Sergio Iván Ravello Arias, Diego Ramírez Muñoz, Jaime Sánchez Moreno, Susana Cardoso, Ricardo Ferreira, Paulo Jorge Peixeiro de Freitas

**Affiliations:** 1 Department of Electronic Engineering, University of Valencia, Avda. de la Universitat, s/n, Burjassot 46100, Spain; E-Mails: sergio.ravelo@uv.es (S.I.R.A.); jaime.sanchez@uv.es (J.S.M.); 2 INESC Microsystems and Nanotechnologies (INESC-MN) and Institute for Nanosciences and Nanotechnologies, R. Alves Redol 9, Lisbon 1000-029, Portugal; E-Mails: scardoso@inesc-mn.pt (S.C.); pfreitas@inesc-mn.pt (P.J.P.F.); 3 INL-International Iberian Nanotechnology Laboratory, Av. Mestre José Veiga, Braga 4715-31, Portugal; E-Mail: ricardo.ferreira@inl.int

**Keywords:** electrical current measurement, magnetoresistance sensor, fractional systems, systems identification

## Abstract

Fractional calculus is considered when derivatives and integrals of non-integer order are applied over a specific function. In the electrical and electronic domain, the transfer function dependence of a fractional filter not only by the filter order *n*, but additionally, of the fractional order α is an example of a great number of systems where its input-output behavior could be more exactly modeled by a fractional behavior. Following this aim, the present work shows the experimental ac large-signal frequency response of a family of electrical current sensors based in different spintronic conduction mechanisms. Using an ac characterization set-up the sensor transimpedance function *Z_t_(if)* is obtained considering it as the relationship between sensor output voltage and input sensing current, $Z_{t}(jf)=\overset{V_{o,\text{sensor}}(jf)}{\;}/\underset{I_{\text{sensor}}(jf)}{\;}$. The study has been extended to various magnetoresistance sensors based in different technologies like anisotropic magnetoresistance (AMR), giant magnetoresistance (GMR), spin-valve (GMR-SV) and tunnel magnetoresistance (TMR). The resulting modeling shows two predominant behaviors, the low-pass and the inverse low-pass with fractional index different from the classical integer response. The TMR technology with internal magnetization offers the best dynamic and sensitivity properties opening the way to develop actual industrial applications.

## 1. Introduction

Fractional calculus is considered when derivatives and integrals of non-integer order are applied over a specific function. In its origin, fractional calculus was a mathematical discipline systematically developed in the beginning and middle of the 19th century by Liouville, Riemann and Holmgren, although there were individual contributions before that (Euler, Lagrange) [1]. At the same time, this emerging field was applied to solve various mathematical problems like linear differential or integral equations. In the last decades, fractional calculus has been a powerful analytical technique to accommodate the actual behavior of a target system in the scientific or engineering domains to a defined set of differential equations, transfer functions or driving-point adpedance functions. In the field of electrochemistry fractional calculus was used to describe more accurately the diffusion processes in electrochemical solutions [2,3] or the equivalent circuit of an electrochemical cell [4,5]. In biochemistry or medicine areas, modeling of biological tissues like skull or intestine have been done with success using the well-known Cole-Cole model. This one considers an impedance in the Laplace domain of the form *Z(s)* = *1/s*^α^, α being non-integer, [6,7]. In botany, the frequency behavior of different fruits and vegetables also have been modeled by fractional electrical impedances [8] or to monitor the microbial growth by means of a signal conditioning circuit based in a sensor described by a fractional impedance model [9]. In the electrical and electronics area, fractional calculus has enjoyed a wide variety of developments. Coils with substantial eddy current and hysteresis losses respond in the frequency domain to a (*jω*)^α^*L* model with α = 0.6, more exactly than the classical α = 1 behavior [10]. In the analogue signal processing field, a great number of studies have been addressed to model fractional capacitors using RC ladders circuits [11–14] or to design fractional order oscillators, differentiators or filters. In the case of oscillators, a significant increase in the oscillation frequency could be reached considering a non-integer exponent (0 < α < 1) in the oscillation capacitance [15,16]. In designing analogue filters one of the most important consequences is that of obtaining slopes in the attenuation band different from multiples of ±20 *n* dB/dec being n the filter order. In this way it could be obtained slopes of ±20 *n* α dB/dec where *α* is the filter fractional order. Additionally, the cut-off frequencies are also *α*-dependent, [17–19]. In industrial electronics fractional controllers have been implemented to stabilize the control loop of switched-mode power converters in solar-powered electrical generation systems [20] or in parameter identification of supercapacitors or lead/acid batteries [21,22].

The transfer function dependence of a fractional filter not only by the filter order n, but additionally, of the fractional order α, is an example of a great number of systems where its input-output behavior could be more exactly modeled by a fractional behavior. Following this aim, the present work shows the experimental ac. large-signal frequency response of a family of electrical current sensors based in different spintronic conduction mechanisms. Using an ac characterization set-up the sensor transimpedance function *Z_t_(if)* is obtained considering it as the relationship between sensor output voltage and input sensing current, $Z_{t}(jf)=\overset{V_{o,\text{sensor}}(jf)}{\;}/\underset{I_{\text{sensor}}(jf)}{\;}$. The study has been extended to various magnetoresistance (MR) sensors based in different technologies like anisotropic magnetoresistance (AMR), giant magnetoresistance (GMR), spin-valve (GMR-SV) and tunnel magnetoresistance (TMR). The obtained experimental results in the ac large-signal characterization process revealed that transimpedance *Z_t_* frequency response is more accurately described by a fractional transfer function behavior.

## 2. Systems with Fractional Representation

### 2.1. Fractional Derivatives and Integrals

Fractional derivatives and integrals (fractional differintegral) are an extension of the classical differential and integral (integer) calculus. A great number of fractional derivatives and integrals definitions have been proposed in the mathematical field, but from an engineering point of view there are specific definitions of special interest.

The forward Grünwald-Letnikov derivative must be considered when studying a fractional system under its steady-state behaviour in the time domain:

(1) $$D_{f}^{\alpha }f(t)=\underset{h\to 0^{+}}{\lim}\frac{\overset{\infty }{\underset{k=0}{\text{∑}}}{\left(-1\right)}^{k}\left(\begin{array}{c} \alpha \\ k \end{array}\right)?f\left(t-kh\right)}{h^{\alpha }}?$$

Being $\left(\begin{array}{c} \alpha \\ k \end{array}\right)$ the binomial coefficients. This definition is based on the incremental ratio and fractional order differences concepts, [23,24].

In the Laplace transform domain the fractional differintegral is much easier to handle. This property is applied to solve problems in fields like biology, medicine or engineering. Applying the bilateral Laplace transform:

(2) $$F(s)=\int_{-\infty }^{+\infty }f(t)\;e^{-st}\;ds$$

to both sides in Equation (1) is it possible to obtain that:

(3) L[Dfαf(t)]=sαF(s),forRe(s)>0

here for *s*^α^ and a cut line in the left half plane [24].

### 2.2. Convolution Integral

Fractional linear-time invariant systems (FLTI) have at the first conception stages the same properties than their previous integer linear-type invariant (ILTI) counterparts. Initial properties like linearity, time invariance are also assumed in the case of FLTI systems [24,25]. Taking them into account, an equivalent behavior is maintained for this type of systems as explained in the following subsections.

**Figure 1.**
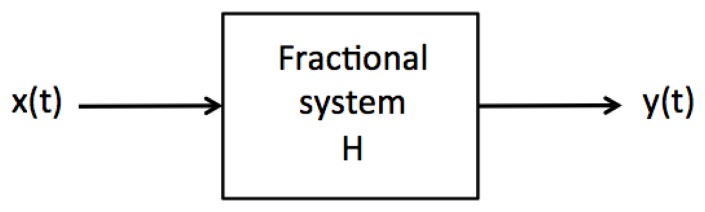
Fractional system representation in the time-domain.

Let *x*(*t*) a continuous-time signal that is to be applied to the input of a fractional system (Figure 1).

The input signal *x*(*t*) could be expressed as a weighted superposition of time shifted impulses:

(4) $$x(t)=\int_{-\infty }^{+\infty }x(\tau )\;\delta (t-\tau )\;d\tau$$

being *δ*(*t*) the impulse function. Let H, the characteristic operator of the FLTI system that is applied to the input signal *x*(*t*) to produce an output signal *y*(*t*):

(5) $$y(t)=H\{x(t)\}=H\{\int_{-\infty }^{+\infty }x(\tau )\;\delta (t-\tau )\;d\tau \}$$

Assuming the linearity of the fractional system, the order of the operator and integration could be interchanged to obtain:

(6) $$y(t)=H\{x(t)\}=\int_{-\infty }^{+\infty }x(\tau )\;H\{\delta (t-\tau )\}\;d\tau$$

If *h*(*t*)≡*H*{*δ*(*t*)} is defined as the output of the system in response to a unit impulse signal and considering the time-invariant property of the fractional system:

(7) H{δ(t−τ)}=h(t−τ)

replacing this result into Equation (6) gives to consider that the fractional system output response *y*(*t*) could be expressed as:

(8) $$y(t)=\int_{-\infty }^{+\infty }x(\tau )\;h(t-\tau )\;d\tau$$

The above expression indicates that the output *y*(*t*) is given as a weighted superposition of system impulse responses time shifted by *t*. The system output, *y*(*t*) is obtained by the convolution integrals of signals *x*(*t*) and *h*(*t*), as in ILTI systems:

(12) $$y(t)\equiv x(t)*h(t)=\int_{-\infty }^{+\infty }x(\tau )\;h(t-\tau )\;d\tau$$

### 2.3. Eigenfunction Property

Considering the commutative property of the convolution operation and taking the input *x*(*t*) in the form *x(t)* = *e^s^*^·^*^t^*, where s is the complex frequency *s*= *σ* + *jω*, the fractional system input response *y*(*t*) will be:

(10) $$y(t)=h(t)*x(t)=\int_{-\infty }^{+\infty }h(\tau )\;x(t-\tau )\;d\tau =\int_{-\infty }^{+\infty }h(\tau )\;e^{s\cdot (t-\tau )}\;d\tau =e^{s\cdot t}\;\int_{-\infty }^{+\infty }h(\tau )\;e^{-s\cdot \tau }\;d\tau$$

The resulting integral is defined as the transfer function *H*(*s*) of the FLTI system. In other words, the input *x(t)* = *e^s^*^·^*^t^* is defined as an eigenfunction of the FLTI system and *H*(*s*) as the responding eigenvalue:

(11) $$y(t)=H(s)\;e^{s\cdot t}$$

On the other hand, as the output system response *y*(*t*) is equivalent to the convolution of *h*(*t*) and *x*(*t*) signals, taking bilateral Laplace transform in Equation (10) leads to:

(12) Y(s)=H(s)X(s)

then, the transfer function of a FLTI system could be expressed as:

(13) $$H(s)=\frac{Y(s)}{X(s)}$$

like in ILTY systems.

### 2.4. Transfer Function

Equation (14) establishes the input-output representation of a FLTI system by means of a differential equation with constant coefficients where *y*(*t*) represents the output and *x*(*t*) the input is assumed to be a continuous-time signal. The constants *a*_1_, *a*_2_, … *a_N_* and *b*_1_, *b*_2_, …, *b_M_* depend on the element values and the internal topology of the system. Its order is determined by the integer numbers *N* and *M* but often *N* ≥ *M* and the order is described using only *N*. In its general format:

(14) $$\sum_{k=0}^{N}\;a_{k}D^{q_{k}}y(t)=\sum_{k=0}^{M}\;b_{k}D^{q_{k}}x(t)$$

where, *a_k_* and *b_k_* are constants coefficients, *q_k_* are assumed to be positive real numbers being the derivative order (with *k* = 0, 1, 2, …) and *D* symbolizes the time derivative operation, $D\equiv \overset{d}{\;}/\underset{dt}{\;}$.

Considering the eigenfunction property, if *x(t)* = *e^st^* then *y(t)*=*H(s) e^−st^*= *e^−st^H(s)*. Replacing *x*(*t*) and *y*(*t*) by their expressions in the fractional differential Equation (14):

(15) $$\sum_{k=0}^{N}\;a_{k}D^{q_{k}}\;\left(e^{st}\;H(s)\right)=\sum_{k=0}^{M}\;b_{k}D^{q_{k}}e^{st}$$

(16) $$\sum_{k=0}^{N}\;a_{k}\;\left(D^{q_{k}}e^{st}\right)\;H(s)=\sum_{k=0}^{M}\;b_{k}D^{q_{k}}e^{st}$$

because the fractional derivative of the exponential function is *D^q_k_^e^st^*= *s^q_k_^e^st^ Re(s)>0* [24], then:

(17) $$\sum_{k=0}^{N}a_{k}s^{q_{k}}e^{st}H(s)=\sum_{k=0}^{M}b_{k}s^{q_{k}}e^{st}$$

and solving for *H*(*s*), it brings an explicit representation of the transfer function for FLTI systems:

(18) $$H(s)=\frac{\overset{M}{\underset{k=0}{\text{∑}}}\;b_{k}s^{q_{k}}}{\overset{N}{\underset{k=0}{\text{∑}}}\;a_{k}s^{q_{k}}}$$

considering *Re* (*s*) > 0 because causal systems are assumed. Again, in ILTI systems the transfer function is a rational quotient between two polynomials of variable *s* with positive real number *q_k_* as exponents and with coefficients *a_k_*, *b_k_* done by the fractional differential equation defined by the system.

The system frequency response *H*(*j*ω) is obtained with *s* = *j*ω and their associated Bode diagrams could be obtained. The main difference compared to ILTI systems is that it could be obtained, in the amplitude asymptotic representation, lines with slopes that have no restrictions about the ±20 dB/dec multiples like integer systems.

In the fractional systems modeling process the above expression for the transfer function *H*(*s*) put complex problems at the time to solve for its poles. In this way, in order to find a transfer function representing the fractional system behavior, a two cases restriction is applied concerning the *q_k_* exponents:

(a) *q_k_* will be irrational numbers but multiples of a given *q*, 0 ≤*q* ≤ 1 or
(b) *q_k_* will be any rational number that could be written in the format *u_k_/v_k_*. In this case, let *u* and *v* the least common multiples of the *u_k_* and *v_k_*, thus, *q_k_* = *k u/v where k and v* are positive integer numbers, so *q_k_* = *k q* with *q* = *1/v*. The coefficients and orders do not coincide necessarily with the previous ones, since some of the coefficients can be zero.

With these considerations the transfer function becomes:

(19) $$H(s)=\frac{\overset{M}{\underset{k=0}{\text{∑}}}\;b_{k}s^{kq}}{\overset{N}{\underset{k=0}{\text{∑}}}\;a_{k}s^{kq}}$$

If the starting point is the transfer function it is possible to obtain, as in the ILTI systems, the system behavior in the time domain using the mathematical techniques offered by the fractional calculus. That is the system response to an input impulse or whatever input signal in the *s*-domain *X*(*s*) by the use of the Laplace transform inversion [24,26].

## 3. Magnetoresistance Sensors

In the last two decades magnetoresistive sensors have attracted great interest from the science and technology point of view. Some authors use the acronym XMR [27,28] to refer to all technologies arising for implementing MR sensors: AMR, GMR and TMR.

**Figure 2.**
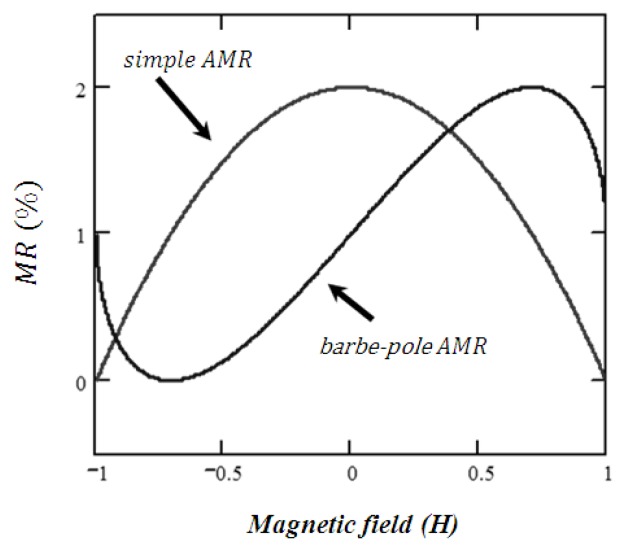
*R*-*H_f_* characteristic of a simple AMR sensor and barber-pole AMR sensor.

AMR technology is the simplest technology in terms of layers and materials. To be consistent with the following MR technologies, an AMR sensor comprises a single-permalloy layer of which magnetic moment is free to rotate due to an external-magnetic field influence *H_f_*. The electrical resistance R is dependent on the angle between the magnetization of this free layer and its biasing current [29]. The *R*-*H_f_* characteristic of a simple AMR sensor is shown in Figure 2. Note that each value of the resistance corresponds to two identical but opposed values of the normalized *H_f_*. This non-linearity is corrected in Figure 2 using the barber-pole geometry [29], in which, the permalloy layer is coated with an Al wire orientated 45° respect to its longitudinal axis therefore creating a biasing field capable to set a linear-like sensor output curve around zero magnetic fields. AMR technology offers a weak MR effect, usually lower than 3%.

**Figure 3.**
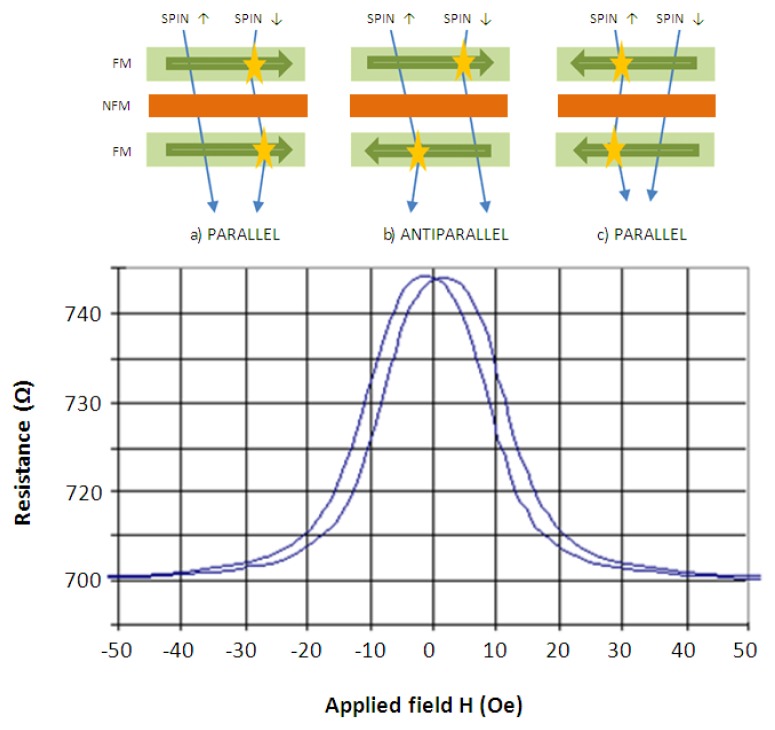
(**Top**) GMR principle illustration; (**bottom**) *R*-*H_f_* characteristic of a GMR sensor.

GMR technology offers sensors with a magnetoresistance variation in percentage terms ranging between 10%–200%. It consists of a multilayer structure where two ferromagnetic layers (FM) are separated by a nanometric non-ferromagnetic conductive layer (NFM). The electrical resistance depends on the scattering suffered by the electrons as a function of its spin (up or down) and the relative orientation of the magnetization between ferromagnetic layers. Its operating principle can be explained using the two-current model described in [30], which proposes that the current through a GMR structure is composed by two channels, one channel for spin-up electron and the other for spin-down electrons. If the orientation of the FM layers is antiparallel, then both channels provide high scattering for spin-up and spin-down electrons. But, in case the configuration of the FM layers is parallel there is a channel with low resistance for electrons either spin up or spin down electrons, depending on *H_f_* direction. Figure 3 represents the *R*-*H_f_* characteristic of a GMR sensor where it can be observed that again it is bi-valued in magnetic field because two values of it (equal in magnitude and opposite in sign) have assigned the same magnetoresistance value. This problem is solved by spin-valve technology (GMR-SV). It consists in a multilayer where one FM layer is free to rotate under an external magnetic field influence meanwhile the other layer is pinned orthogonally [31]. The GMR-SV effect is lower than GMR, but a change equal to 6%–20% is still greater than AMR technology.

**Figure 4.**
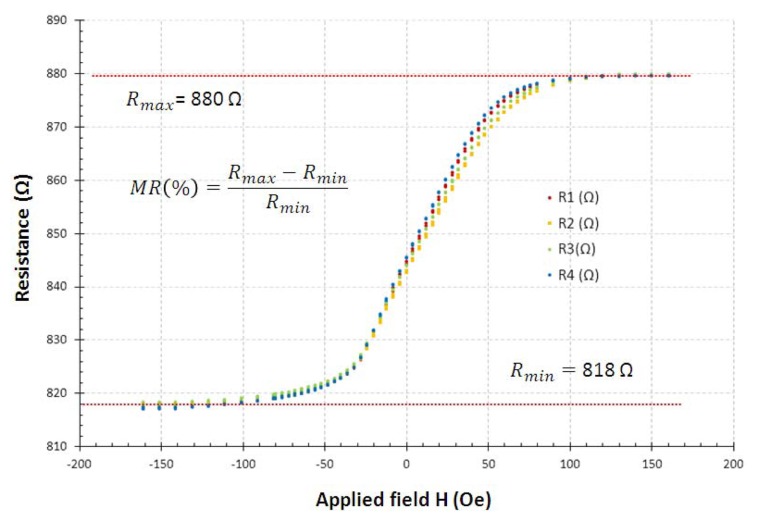
*R*-*H_f_* characteristic of the spin valve sensor used in this work (MR = 7%) [30].

Figure 4 shows the *R*-*H_f_* characteristic for a GMR-SV element. In this case, the sensor were microfabricated at INESC-MN Lisbon, with the following structure (thickness in Å): Si/Al_2_0_3_ 500//Ta 30/Ni_80_Fe_20_ 30/Mn_77_Ir_23_ 60/Co_80_Fe_20_ 30/Cu 19/Co_80_Fe_20_ 25/Ni_80_Fe_20_ 25/Ta 20/Ti_10_W_90_(N2) 50, free and pinned layers easy axis were set orthogonally in order to enhance a linear transfer curve, [32,33].

**Figure 5.**
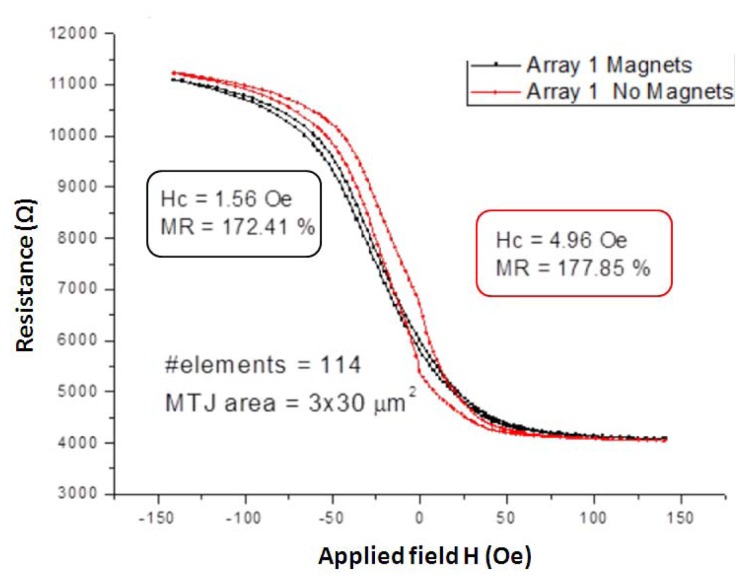
*R*-*H_f_* characteristic of the TMR sensor used in this work (177% MR), [36].

TMR technology provides the highest MR ratios (up to 500%) [34], its structure derives from GMR structures where the NFM conductive layer is replaced by an NFM insulating layer (MgO, Al_2_O_3_, for example). The electrons flow through the insulating layer by means of tunneling effect defined by quantum mechanics. They have strong likelihood to cross the insulating layer when the magnetization vectors of adjacent FM layers are in parallel alignment and low probability when they are in antiparallel alignment. These two states correspond respectively to low and high electrical resistance state. Although the variation of resistance is due to different physical principles, GMR and TMR structures exhibit a similar *R*-*H_f_* characteristic curve, and are based on the same principles as the spin valve technology to obtain a linear response. TMR main drawback is the presence of hysteresis in its *R*-*H_f_* characteristic, however this can be minimized using integrated permanent magnets [35]. Figure 5 shows the *R*-*H_f_* characteristics corresponding to two TMR sensors one with and the second without biasing magnets. These characteristics belong to a MTJ stack based in MgO that was deposited at the International Iberian Nanotechnology Laboratory (INL, Braga, Portugal) in the Timaris sputtering system, with a layer structure of: Si/2000 SiO_2_/50 Ta/500 CuN/50 Ta/500 CuN/50 Ta/50 Ru/75 IrMn/20 CoFe/8.5 Ru/26 CoFeB/10 MgO/30 CoFeB/2.1 Ta/160 NiFe/100 Ta/300 CuN/70 Ru/150 TiW(N) (thickness Å). The permanent magnets were then implemented with CoCrPt thin film elements, [36], patterned after the sensor microfabrication.

XMR sensors have been applied as hard disk read heads, electronic compasses, displacement transducers, encoders and proximity switches, between others. In this paper the XMR sensors are used for electrical current measurement, their main advantages are the inherent galvanic isolation, the level of integration and the ability to measure dc and ac currents. The experimental results of this work are focused in comparing the AMR, GMR and TMR ac large-signal frequency responses. The sensors characterized in this work were set in full or half Wheatstone bridge configurations.

## 4. Experimental Procedure and Methodology

**Figure 6.**
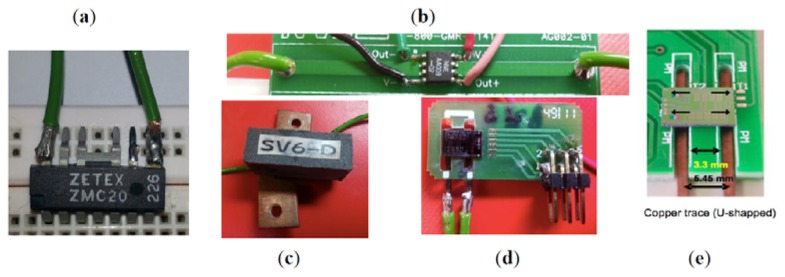
Actual appearance of the tested MR sensors. (**a**) AMR sensor (ZMC20); (**b**) GMR sensor (AA003-02); (**c**) GMR-SV sensor (INESC-MN, Lisbon, Portugal); (**d**) TMR-without magnets (INL, Braga, Portugal) and (**e**) TMR-with magnets (INL).

The main goal of the present work was modeling using fractional calculus, the transimpedance function *Z^t^(if)* in various MR current sensors considering it as the relationship between sensor output voltage and input sensing current, $Z_{t}(jf)=\overset{V_{o,\text{sensor}}(jf)}{\;}/\underset{I_{\text{sensor}}(jf)}{\;}$. The target group was configured by two commercial MR sensors: the ZMC20 part, based on the AMR technology, the AA003-02 part, based on GMR technology and three specific MR sensors micro-fabricated at the INESC-MN and INL facilities: one based on GMR-SV technology [32], and the two last based on TMR technology (without and with internal magnetization) [36–38]. Figure 6 shows the sensors, each one attached over an appropriated printed circuit board or wire in order to sense the current through a copper trace or conductor.

The ZMC20 sensor has inside the plastic package an internal current carrying conductor (Figure 6a top) this arrangement is different from the others MR sensors where the conductor is out of the sensor package. In the case of the AA003-02 part there is a copper trace placed in the printed circuit board and below the sensor (Figure 6b top). The GMR-SV and TMR sensors have a U-shaped copper trace conductor under the sensor active area (Figures 6c–e respectively).

**Figure 7.**
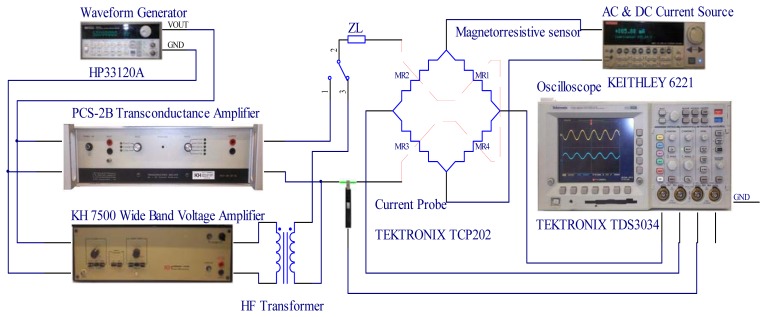
Experimental set-up to test the sensors transimpedance AC large-signal frequency response.

**Figure 8.**
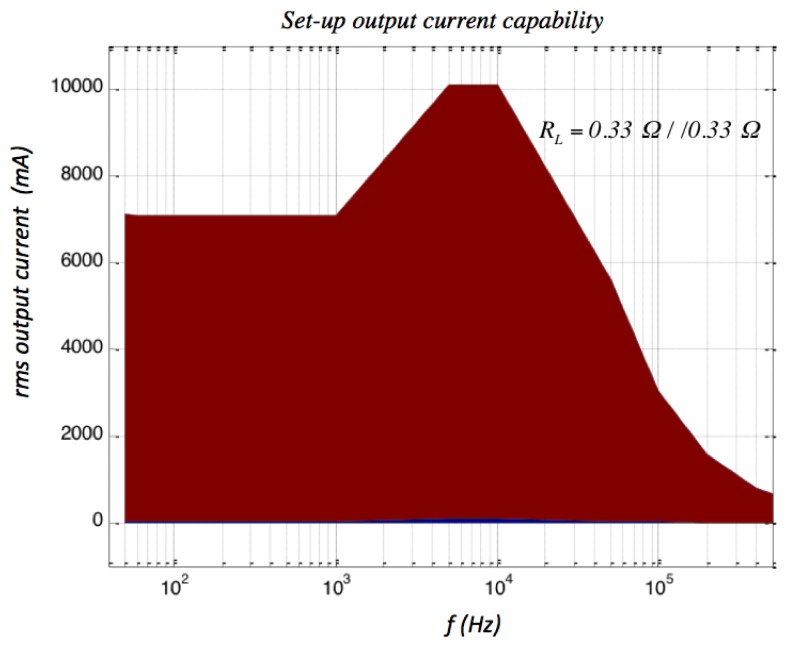
High-frequency set-up output current capability.

**Figure 9.**
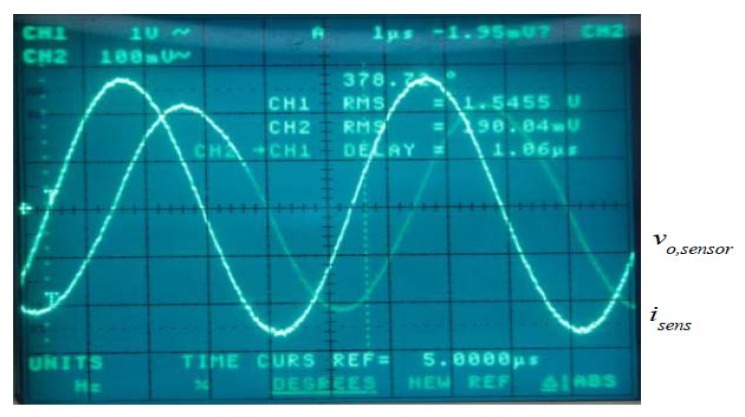
TMR sensor-without magnets output voltage (CH2) *vs.* sensing current (CH1), *I_rms_* = 1.54 A, *f* = 200 kHz.

In all the cases a frequency sweep was selected for a sensor sine wave input current using a high level rms large signal value in amplitude. Figure 7 shows the experimental set-up used to obtain the ac transimpedance frequency response. All the current sensors were energized by a 2 mA constant current source (mod. 6221, Keithley, Cleveland, OH, USA) and the current to be measured by them was generated by two voltage-controlled amplifiers. From a practical point of view it was difficult to have a unique equipment generating in good conditions and simultaneously the current amplitude and frequency needed by the test requirements. Consequently, the whole test was divided in two: the low and the high frequency tests. The low-frequency test was done using a transconductance amplifier (3200 PCS-2B, Krohn-Hite, Brockton, MA, USA) because it has a low cut-off frequency response. In the case of PCS-2B model a peak current amplitude of 10 A was guaranteed between the 40 Hz–1 kHz band. On the other hand, the high frequency test required an equipment prepared to reach the highest possible upper frequency response. In this work the sensing current was generated by a high-frequency transformer with a 21:1.1 turns ratio [38] connecting in its primary side a wide band voltage amplifier (Krohn Hite 7500) with a dc-to-1 MHz frequency response. The ac test was carried out up to a 400 kHz frequency because this limit was imposed by the transformer frequency response limitations. Figure 8 shows the output current capability of the high frequency test (wide band voltage amplifier and voltage transformer loaded with 0.165 Ω). This equipment is able to generate rms current levels of 10 A in a reduced frequency interval (5–10 kHz) but it is capable to reach easily 0.8 A_rms_ at 400 kHz. In both tests the reference dc voltage signal was generated by a waveform generator (Agilent 33120A). The sensor output voltage is a differential signal and in all the cases was measured by an oscilloscope (TDS3034, Tektronix, Beaverton, OR, USA) using two identical and compensated 1:1 voltage probes using the difference mode of the oscilloscope. A differential voltage probe was not used in the test because this would cause additional gain errors and phase shifts. The current measurement was done by the Tektronix TCP202 current probe and the sensor transimpedance *Z_t_*(*jf*) function was obtained taking readings of the rms input current and output sensor voltage and the phase shift between both signals (Figure 9). The sensors studied were submitted to input rms current values between 100 mA and 3.6 A for frequencies of 50 Hz, 60 Hz, 200 Hz, 500 Hz, 1 kHz, 5 kHz, 10 kHz, 50 kHz, 100 kHz and 200 kHz and with 0.8 A_rms_ for 400 kHz frequency.

## 5. Experimental Results, Modeling and Discussion

For each MR sensor the transimpedance *Z_t_*(*jf*) function in the frequency domain was obtained both in amplitude and phase formats (Bode diagrams) from the expressions:

(20) $$\left|Z_{t}(jf)\right|=\frac{\text{rms}\;(v_{o,\text{sensor}})}{\text{rms}\;(i_{\text{sens}})}$$

(21) $$\text{phase}\left[Z_{t}(jf)\right]=\text{phase}\;(v_{o,\text{sensor}})-\text{phase}\;(i_{\text{sens}})$$

**Figure 10.**
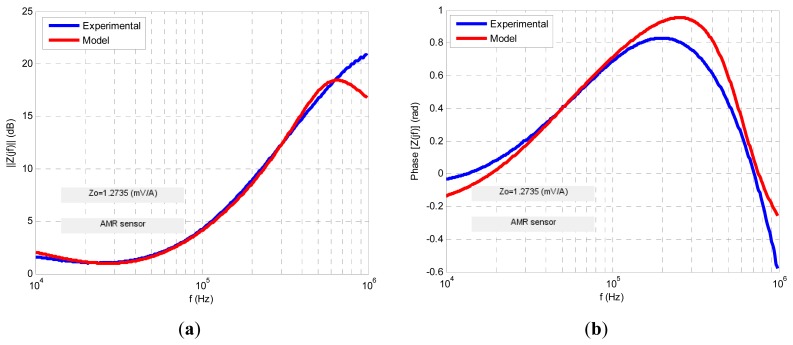
AMR sensor ac large-signal fractional modeling.

**Figure 11.**
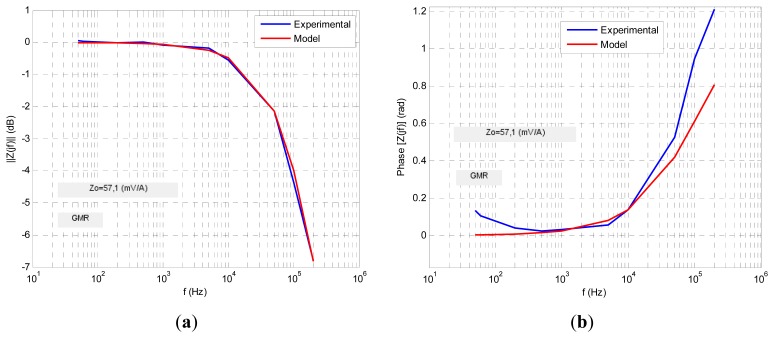
GMR sensor ac large-signal fractional modeling.

**Figure 12.**
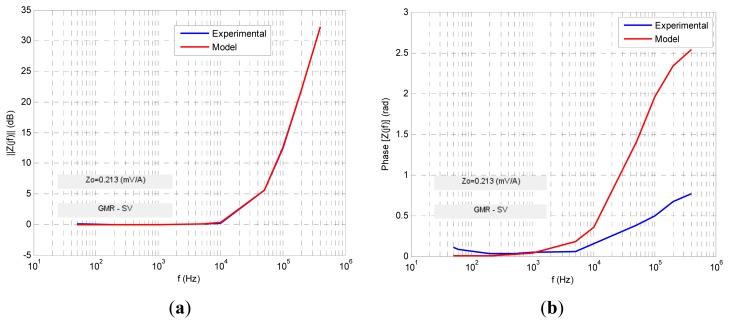
GMR-SV sensor ac large-signal fractional modeling.

**Figure 13.**
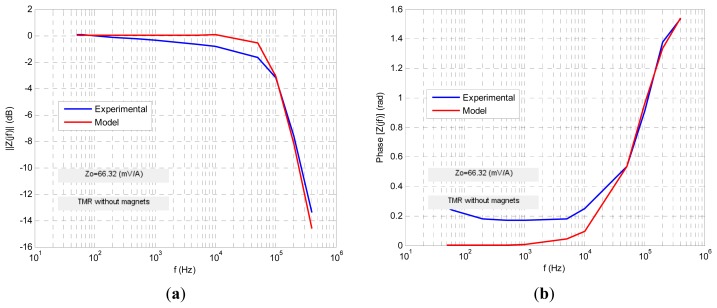
TMR sensor without internal magnetization ac large-signal fractional modeling.

**Figure 14.**
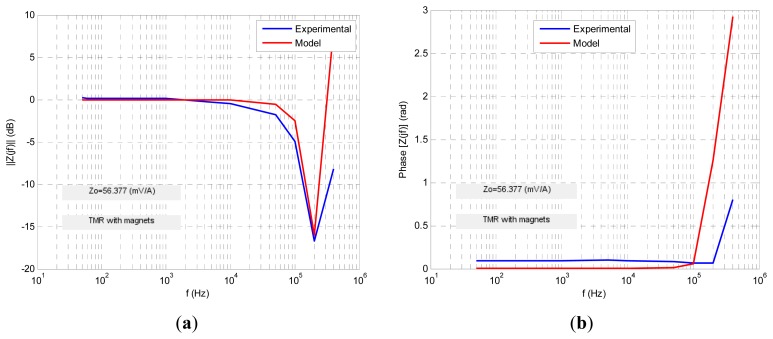
TMR sensor with internal magnetization ac large-signal fractional modeling.

Four transfer functions were fitted numerically using Matlab software to fit the experimental ac response to a least square algorithm to minimize the relative error function. The results are shown in the following figures. For each sensor its normalized transimpedance ac large-signal frequency response is obtained both in magnitude and phase (blue lines) and at the same time is depicted (in red line) the response of the proposed fractional model. The following figures (Figures 10, 11, 12, 13 and 14) and tables (Tables 1, 2, 3 and 4) summarize the fractional model parameters obtained.

With the goal to model the obtained experimental response (Figure 10) a two poles and two zeros transimpedance function was obtained as the most appropriate with poles located in *f*_p1_ = 10 kHz and *f*_p2_ = 600 kHz and zeros in *f*_z1_ = 38 kHz and *f*_z2_ = 245 kHz with fractional orders of α_1_ = 0.59, α_2_ = 1.5, β_1_ = 0.95 and β_2_ = 0.85 respectively. The obtained low frequency transimpedance gain *z*_o_ was 1.3 mV/A. Equation (22) summarizes the proposed model.

(22) $$z(jf)=z_{o}\frac{\left({\left(j\frac{f}{f_{z1}}\right)}^{\beta 1}+1\right)\left({\left(j\frac{f}{f_{z2}}\right)}^{\beta 2}+1\right)}{\left({\left(j\frac{f}{f_{p1}}\right)}^{\alpha 1}+1\right)\left({\left(j\frac{f}{f_{p2}}\right)}^{\alpha 2}+1\right)}$$

In this case a −3 dB frequency of *f*_-3dB_ = 116 kHz was obtained. Experimental measurements and modeling process revealed that GMR and TMR-without magnets sensors offer a fractional low-pass behavior, the first with a fractional index *α* less than unity and the second greater than it (Figures 11 and 13, Tables 1 and 3). Additionally both sensors have an ac large-signal −3 dB bandwidth around 70–100 kHz and similar *z_o_* dc sensitivity. On the other hand GMR-SV and TMR-with magnets manifest an inverse fractional low-pass behavior with fractional index *α* slightly less than 2 (Figures 10, 12 and 14 and Tables 2 and 4). Note the presence of a resonance region in the TMR-with magnets response (Figure 14) as a consequence of its fractional index approximation to the α = 2 value in conjunction with its characteristic frequency *f*_c_. As far as the experimental set-up can (0.8 A_rms_ with *f* = 400 kHz) the measurements revealed an increasing transfer function with frequency in the high frequency zone. Probably using an improved testing experimental set-up the transfer function will be limited in magnitude but this behaviour cannot be shown yet. Nevertheless unless the transfer function is not limited in the high frequency region a compensation of this not-limited magnitude can be done connecting in cascade at the sensor output a differential low-pass filter properly designed.

**Figure 15.**
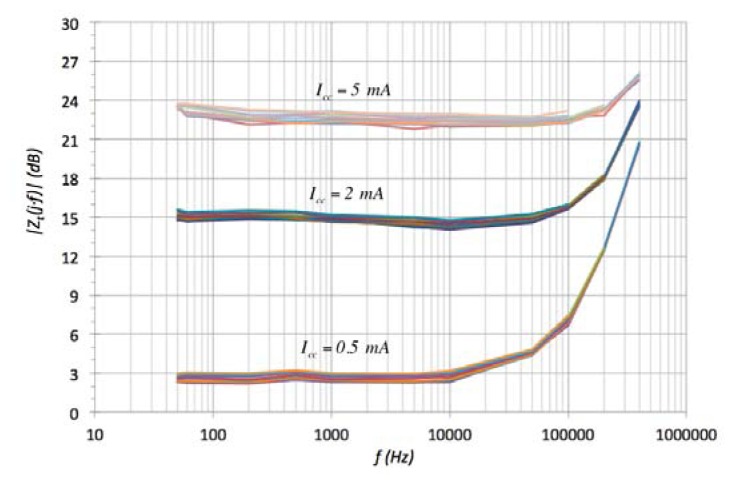
Dynamic behavior dependence with *I_cc_* in TMR sensor without permanent magnet linearization.

The AMR sensor responds to a more complex model with two zeros and two poles with their fractional index. The widest ac large-signal −3 dB frequency band are offered by the TMR technology (without and with magnets) providing at the same time higher *z_o_* dc sensitivity (Tables 3 and 4). These properties give the TMR technology an appropriate MR sensor to implement actual industrial applications. Moreover, it is possible to extend the practical frequency band applying the extension technique described in [39]. One effect observed in TMR-with magnet sensor was the variation of the bandwidth with the power supply bias current *I_cc_*. Figure 15 shows how the corner frequency of the transimpedance function is shifted to higher frequency region as the sensor constant current power supply increases. Nevertheless a trade-off must be reached between bandwidth and power consumptions in order to optimize the sensor use.

## 6. Conclusions

The present work has been an attempt to model the ac large-signal frequency response of different MR current sensor technologies using fractional calculus. From the experimental measurements obtained by a specific high-current wide bandwidth set-up different fractional transimpedance functions have been fitted. The resulting modeling shows two predominant behaviors: the low-pass and the inverse low-pass with fractional index differ from the classical integer response. The TMR technology with internal magnetization (permanent magnet biasing stabilization) offers the best dynamic and sensitivity properties, opening the way to develop actual industrial applications.

This work was supported in part by the Spanish Ministry of Economics and Competitivity under the AYA2012-37444-C02-01 project by the Generalitat Valenciana under the Prometeo/2012/044 project and by the 217152-312630 grant of the Consejo Nacional de Ciencia y Tecnología (CONACYT México). INL acknowledges partial funding from the ON2 project from PO-Norte. INESC-MN acknowledges FCT funding through the Instituto de Nanociência e Nanotecnologia (IN) Associated Laboratory.

## Figures and Tables

**Table 1.** GMR sensor ac large-signal fractional modeling parameters.

***Z****_t_***(*jf*)**	**α**	***f****_c_*** (kHz)**	***Z****_o_*** (mV/A)**	***f*−***_3dB_*** (kHz)**
$\frac{z_{o}}{1+{\left(j\frac{f}{f_{c}}\right)}^{\alpha }}$	0.8	105.6	57.1	72.2

**Table 2.** GMR-SV sensor ac large-signal fractional modeling parameters.

***Z****_t_***(*jf*)**	**α**	***f****_c_*** (kHz)**	***Z****_o_*** (mV/A)**	***f*−***_3dB_*** (kHz)**
$z_{o}\left[1+{\left(j\frac{f}{f_{c}}\right)}^{\alpha }\right]$	1.8	48.3	0.21	25.5

**Table 3.** TMR sensor without internal magnetization ac large-signal fractional modeling parameters.

***Z****_t_***(*jf*)**	**α**	***f****_c_*** (kHz)**	***Z****_o_*** (mV/A)**	***f*−***_3dB_*** (kHz)**
$\frac{z_{o}}{\left(1+{\left(j\frac{f}{f_{c}}\right)}^{\alpha }\right)}$	1.1	86	66.3	99

**Table 4.** TMR sensor with internal magnetization ac large-signal fractional modeling parameters.

***Z****_t_***(*jf*)**	**α**	***f****_c_*** (kHz)**	***Z****_o_*** (mV/A)**	***f*−***_3dB_*** (kHz)**
$z_{o}\left[1+{\left(j\frac{f}{f_{c}}\right)}^{\alpha }\right]$	1.9	204	56.4	108
